# Domestic violence patterns in postpartum women who delivered during the COVID-19 pandemic

**DOI:** 10.1590/1980-549720240022

**Published:** 2024-04-19

**Authors:** Luciano Lima Correia, Márcia Maria Tavares Machado, Anya Pimentel Gomes Fernandes Vieira-Meyer, David Augusto Batista Sá Araújo, Emanuel de Assis Bertulino Martins Gomes, Anyelle Barroso Saldanha, Rita de Cássia Rebouças Rodrigues, Yuri Valentim Carneiro Gomes, Márcia Caldas Castro

**Affiliations:** I Universidade Federal do Ceará, Department of Community Health – Fortaleza (CE), Brazil.; II Fundação Oswaldo Cruz, Family Health – Eusébio (CE), Brazil.; III Universidade Federal do Ceará – Fortaleza (CE), Brazil.; IV Harvard T. H. Chan School of Public Health, Department of Global Health and Population – Boston (MA), USA.

**Keywords:** Domestic violence, Maternal health, Postpartum period, Cohort studies, Epidemiologic factors, Violência doméstica, Saúde materna, Período pós-parto, Estudos de coorte, Fatores epidemiológicos

## Abstract

**Objective:**

To longitudinally assess domestic violence (DV) during the postpartum period, identifying types, patterns and determinants of DV, according to mothers’ reports in Fortaleza, Brazil.

**Methods:**

Data from the Iracema-COVID cohort study interviewed at home mothers who gave birth in the first wave of COVID-19, at 18 and 24 months after birth. Patterns of reported DV were classified as follows: no DV, interrupted DV, started DV and persistent DV. Adjusted multinomial logistic regressions were used to assess factors associated with persistent DV.

**Results:**

DV was reported by 19 and 24% of the mothers at 18 and 24 months postpartum, respectively, a 5 percentage points increase. Persistent DV was present in 11% of the households in the period. The most frequent forms of DV were verbal aggression, reported by 17–20% of the mothers at 18 and 24 months, respectively; drunkenness or use of drugs at home, present in 3–5% of the households; physical aggression, reported by 1.2–1.6% of the mothers. Households with two or more forms of DV increased from 2 to 12% in the period. Adjusted factors associated with persistent DV were maternal common mental disorder, family headed by the mother and head of family’s poor schooling. Food insecurity was associated with starting DV.

**Conclusion:**

Prevalence of DV was considerably high in the postpartum period. DV prevention policies should rely on improving care to women’s mental health; preventing food insecurity; and fostering the educational level of young people of both sexes.

## RESUMO

**Objetivo:**

Avaliar a violência doméstica (VD) longitudinalmente no período pós-parto, identificando tipos, padrões e determinantes de VD, segundo relatos de mães em Fortaleza, Brasil.

**Métodos:**

O estudo de coorte Iracema-COVID entrevistou em casa mães que pariram na primeira onda de COVID-19, aos 18 e 24 meses após o parto. Os padrões de VD relatados foram classificados da seguinte forma: VD inexistente, VD interrompida, VD iniciada e VD persistente. Regressões logísticas multinomiais brutas e ajustadas com variância robusta foram utilizadas para avaliar os fatores associados à VD persistente.

**Resultados:**

A VD foi relatada por 19–24% das mães aos 18 e 24 meses pós-parto, respectivamente, mostrando um aumento de 5 pontos percentuais. Em 11% dos domicílios a VD persistente esteve presente no período. As formas de VD incluíram agressão verbal, relatada por 17–20% das mães; embriaguez ou uso de drogas em casa, presente em 3-5% das residências; agressão física, relatada por 1,2–1,6% das mães. Residências com duas ou mais formas de VD aumentaram de 2 para 12% no período. Fatores de risco ajustados associados à VD persistente foram: transtorno mental comum materno, família chefiada pela mãe e baixa escolaridade do chefe de família. Insegurança alimentar esteve associada à VD iniciada.

**Conclusão:**

A prevalência de VD foi consideravelmente alta no período pós-parto. Políticas de prevenção de VD devem se basear em intervenções que visem melhorar a atenção à saúde mental das mulheres; combater a insegurança alimentar; e promover o nível educacional de jovens de ambos os sexos.

## INTRODUCTION

It is estimated that, globally, one out of three women aged 15 or older have experienced some form of physical, psychological, or sexual violence in their lifetime, most often perpetrated at home by their partners^1^. Even when violence is mutual, women tend to be more vulnerable^2^. While domestic violence (DV) occurs in all social classes, ethnicity, religion, or culture, vulnerable and historically marginalized populations, such as black and/or poor women, seem to sustain longer effects^3^. DV happens in many forms and varying degrees of severity, being widely recognized as a severe human rights violation, with notable public health implications, bringing extensive consequences not only for the women but also for their siblings and family.

Brazil has been recognized as one of the countries with the highest rates of violence in the world. A report from the Brazilian Public Security Forum shows that although general homicide rates have dropped in recent years, female homicides increased. As a whole, all forms of violence against women increased in 2022, with 18 million women reporting at least one episode of violence^4^. Juridically the country has recognized the problem, passing in 2006 the *Maria da Penha* law for the protection of DV victims from their perpetrators, and the feminicide law that increased the severity of penalty in cases where the woman is killed. Despite the legislation, women still fall victim to DV and avoid denouncing their aggressors due to fear of retaliation, lack of confidence in the protective measures, and economic dependency on partners, among others^4^.

Several studies also show that DV is particularly high in the perinatal period, with higher rates of DV during pregnancy in low- and middle-income countries^5-7^, while in high-income countries the greater rates of DV are found in the postpartum period^8^. Despite of being a middle-income country, Brazil has higher rates of DV in the post-partum period, like the high-income countries.

DV is significantly associated with multiple factors, including age at marriage, parity, family size, family history of violence, low educational and/or economic status, alcohol and illicit drug consumption, household food insecurity, women who disobey their partner, women who believe in women’s right, and women with decision-making power^5-7,9^. However, few studies have analyzed DV from a longitudinal perspective. Here we address this gap. We utilize data from the Iracema-COVID Cohort study, which follows women who were pregnant and delivered during the COVID-19 pandemic, to assess determinants of DV, and to characterize patterns of persistent DV over time.

## METHODS

This was a longitudinal study of DV in households of mothers who gave birth during the COVID-19 pandemic, followed up by the Iracema-COVID, a prospective cohort study carried out in Fortaleza, the capital city of the state of Ceará, Northeastern region of Brazil^10^. The study was approved by the Research Ethics Committee in Brazil (number 31190420.4.0000.5054).

Fortaleza had an estimated population of approximately 2.7 million inhabitants in 2020, and a human development index (HDI) of 0.754. The city is divided into 121 neighborhoods, distributed in six health administrative regions. Around three-quarters of the population rely on the public national health system (called SUS) and one-quarter uses private insurance, with very few affording to pay out of pocket for medical services. Hospital deliveries reach about 98% of the city’s births, of which 75% are carried out in public facilities, free of charge.

Iracema-COVID sample was designed to be representative of Fortaleza, considering the population size of the six administrative regions. The sample size was calculated (n=352) to detect a prevalence of maternal common mental disorder (Cohort’s main outcome)^11^, with a margin of error of 5 percentage points, and a 95% confidence interval. For sampling purposes, we used data from the Brazilian Live Birth Information System (SINASC) of July and August 2020. Mothers who lived in Fortaleza gave birth in public hospitals (about 75% of all births), and had complete address information, were eligible to participate. Women that gave birth in private hospitals were deemed ineligible due to not having their contact information available in public records. Out of 4,840 mothers that gave birth in July and August 2020, 3,567 were eligible for the study. Of those, 724 women were randomly sampled (as the desired sample size was 352, we selected 372 additional women in anticipation of refusals to participate and occasional problems with wrong or changed addresses). Sample calculations were done using the GSAMPLE module in Stata (StataCorp. 2019).

All 724 women had at least three contact attempts and, of them, 351 agreed to participate in the baseline study, being interviewed face to face at home at six months postpartum^10^. At subsequent survey rounds (12, 18, and 24 months after birth), cohort participants were tracked and contacted by trained interviewers. In the two survey rounds in which the DV issue was studied, at 18 and 24 months, 331 and 322 mothers, respectively, agreed to participate. The interviews took place as privately as possible in the homes, without the presence of the partners.

At 18 and 24 months postpartum mothers were asked whether in the previous six months they had experienced some episode of violence at home, either as a victim or as a perpetrator. The reported DV episodes referred not only to those involving the intimate partner, but also another family member. Thus data on DV relied on mothers’ reports in two moments over the second year after birth. More specifically, DV was explored through five dichotomous variables to denote the presence or absence of DV in the previous six months. In the first question, women were broadly asked whether there had been ‘fights, arguments or other types of violence at home’. The subsequent questions were more specific and asked if, in the previous six months, each of these types of problems had occurred at home: verbal aggression against the woman (argument, naming, cursing); physical aggression against the woman (push, beat, kick, punch); partner arriving drunk or drugged; and threatening to expel mother from home. A positive answer to any of the five items above qualified the respondent as exposed to some form of DV in the previous six months, e.g., from 12 to 18 months after birth. The same set of DV questions was repeated at 24 months postpartum, thus covering a recall period of twelve months (12 to 24 months postpartum).

The outcome longitudinal variable expressing the patterns of DV over the period covered by the two survey rounds comprised four categories as follows:

a) No DV — when mothers did not report any form of DV either in the third or the fourth rounds.
b) Interrupted DV — when mothers reported at least one form of DV in the third, but not in the fourth round.
c) Started DV — when mothers did not report any form of DV in the third, but reported at least one form of DV in the fourth round.
d) Persistent DV — when mothers reported at least one form of DV in both rounds.

To assess potential factors associated with DV, maternal and family characteristics were selected through a conceptual model. At the model’s distal level, the predictors included socioeconomic status, monthly family income, cash transfers, food insecurity, and administrative district of residence. At an intermediate level, we considered the mother’s age, self-reported skin color, schooling, marital status, smoking, and alcohol consumption. Lastly, at a proximal level, we included living without the child’s biological father and maternal depressive symptoms.

Maternal age was grouped into three categories: 18 to 29, 30 to 39 ³40 years. Geographically, mothers were classified according to residence in the six administrative districts. Self-reported maternal skin color was categorized as white, black, and brown. Schooling was assessed through the mother’s number of years of formal education (0 to 8, 9 to 11, and >11). Smoking and alcohol consumption were assessed as binary Yes/No questions. Maternal depressive symptoms were assessed by the Self-Report Questionnaire (SRQ-20), a 20-item self-report screening tool developed by the WHO to detect psychological distress. The SRQ-20 was validated for application to the Brazilian population using a cut-off point of eight or more as a positive indicator of morbidity with 83% sensitivity and 80% specificity. Food insecurity was assessed through the Brazilian Food Insecurity Scale (EBIA).

Iracema-COVID’s third survey round (18 months after birth) corresponded to the beginning of the third wave of the COVID-19 pandemic in January 2022, while the fourth survey round comprised the period from August to October 2022. All survey rounds utilized standardized questionnaires. Verbal informed consent was obtained from all participants.

Descriptive statistics of maternal characteristics were calculated for each survey round. Tests of differences between the characteristics of the two survey rounds were carried out using a chi-square test. The proportion of participants reporting DV in each survey round, considering a recall period of six months, was examined. The difference in proportions of DV reporting was calculated, by comparing the third and fourth survey rounds using McNemar’s test for paired data^12^. Crude and adjusted multinomial logistic regressions with robust variance were performed to estimate odds ratios, and their respective 95% confidence intervals (95% CI), for mothers who stopped, started, and persistently reported DV, using mothers who did not report DV as a reference, as well as identifying associations between reported DV and the investigated predictors. Statistical analyses were performed in STATA version 16.1 (StataCorp. 2019. Stata Statistical Software: Release 16. College Station, TX: StataCorp LP).

## RESULTS

A total of 331 e 322 mothers were followed up at 18 and 24 months postpartum, respectively, in the Iracema-COVID Cohort Study. Of them, 321 were followed up in both rounds. About one quarter of mothers had between 18 and 24 years of age, only 21% had not reached high school, and about half had a job. Family income and participation in cash transfer programs decreased significantly from 18 to 24 months postpartum (Table 1).

**Table 1:** Distribution of main maternal and family study sample characteristics at 18 and 24 months postpartum. Iracema-COVID Cohort Study. Fortaleza (CE), Brazil, 2021–2022.

Characteristics	3^rd^ round: 12 to 18 months after birth*	4^th^ round: 18 to 24 months after birth*	p-value
	n (%)	n (%)	
Age (years)			
<25	89 (26,9)	78 (24,2)	0,435
25 or above	242 (73,1)	244 (75,8)	
Marital status			
Single	107 (32,3)	97 (30,1)	0,544
Married	133 (40,2)	195 (60,6)	
Other	91 (27,5)	30 (9,3)	
Schooling (years)			
<8	70 (21,2)	68 (21,1)	0,992
≥8	261 (78,8)	254 (78,9)	
Working mother			
Yes	172 (51,9)	145 (44,9)	0,076
No	159 (48,1)	178 (55,1)	
Common mental disorder:			
Yes	277 (83,7)	256 (79,5)	0,167
No	54 (16,3)	66 (20,5)	
Monthly family income			
<1 MW^†^	64 (19,3)	86 (26,6)	0,028
1–2 MW	187 (56,5)	172 (53,3)	
3 MW or above	78 (23,6)	64 (19,9)	
Cash transfer			
Yes	212 (64,1)	181 (53,8)	0,041
No	119 (35,9)	141 (44,2)	
Food insecurity:			
Severe or moderate	187 (56,5)	172 (53,3)	0,275
Mild or no FI	78 (23,6)	64 (19,9)	

*3^rd^ round: n=331; 4^th^ round: n=322;

^†^MW: mininum wage.

DV was reported by 19 and 24% of the mothers at 18 and 24 months postpartum, respectively, showing a 26% increase in the period. Verbal aggression was the most common form of DV, reported by 17% of the mothers in the 3^rd^ and 20% in the 4^th^ survey rounds. Drunkenness and/or illicit drug abuse at home was the second most frequent form of DV, present in 3–5% of the households. Physical aggression was reported by only 1.2 and 1.6% of the mothers in the 3^rd^ and 4^th^ survey rounds, respectively. The proportion of households with two or more forms of harassment increased from 2% at 18 months to 12% at 24 months postpartum (Table 2).

We found that in 11% of the households DV was persistently present during the second year postpartum, while 68% of the mothers did not report the presence of any form of DV in the household (Table 3). Among mothers who experienced changes in the pattern of DV, 8% experienced cessation of DV at home, while 13% were exposed to a DV ramping up. In Table 3 the difference between such changes in the pattern of DV (difference of proportion) indicates a 4.98% increase (p=0.048) in DV reports by mothers from the 3^rd^ (18 months) to the 4^th^ survey round (24 months).

**Table 2:** Prevalence of domestic violence in the 3^rd^ and 4^th^ survey rounds. Iracema-COVID Cohort Study. Fortaleza (CE), Brazil, 2021–2022.

Type of domestic violence	3^rd^ round: 12 to 18 months after birth		4^th^ round: 18 to 24 months after birth	
	n	%	n	%
Overall domestic violence				
Yes	63	19.04	77	23.91
No	268	80.97	245	76.09
Verbal aggression*				
Yes	56	16.97	65	20.25
No	274	83.03	256	79.75
Physical agression^†^				
Yes	4	1.22	5	1.55
No	326	98.79	317	98.45
Drunkness or use of drugs at home				
Yes	9	2.72	15	4.66
No	321	97.28	307	95.34
Kicking off home				
Yes	9	2.72	10	3.11
No	321	97.28	312	96.89
Unspecified domestic violence				
Yes	20	6.06	34	10.56
No	310	93.94	288	89.44
Number of types of violence in the same household				
0	268	80.97	245	76.09
1	56	16.92	40	12.42
2	3	0.91	28	8.70
3 or more	4	1.21	9	2.80

*Verbal aggression, including arguments, name-calling, etc.;

^†^Physical aggression, including beating, pushing, kicking, punches, etc.

**Table 3:** Domestic violence in the 3^rd^ e 4^th^ survey rounds. Iracema-COVID Cohort Study. Fortaleza (CE), Brazil, 2021–2022.

Survey rounds		4^th^ survey round: 18 to 24 months after birth, n (%)		
		No	Yes	Total
3^rd^ survey round: 12 to 18 months after birth, n (%)	No	220 (68.0)	41 (13.4)	261 (81.3)
	Yes	25 (7.8)	35 (10.9)	60 (18.7)
	Total	245 (76.3)	76 (23.7)	321 (100)

Difference in proportions (McNemar’s Test) and 95%CI=4.98% (-0.26; 10.23), p-value=0.048.

Factors associated with reported DV were initially identified in the bivariate analysis (Table 4). Maternal factors included: common mental disorder (CMD), according to the SRQ20 scale, with a 4-fold greater probability of persistent DV (p<0.001) and elementary level maternal schooling, particularly uncompleted level (p=0.050). Family-related factors included: food insecurity (FI), with mothers with moderate to severe FI showing twice the risk of starting DV or persistent DV as compared to those experiencing mild FI (p=0.004); the mother as the head of the household doubled the probability of persistent DV, as compared to households headed by the partner or grandparents (p=0.009); a head of household with either poor schooling or university degree was significantly associated to persistent DV (p=0.006); and participating in the government cash transfer program (*Bolsa Família*) also doubled the probability of persistent DV (p=0.052).

**Table 4:** Maternal and family features associated with domestic violence during the second year postpartum. Iracema-COVID Cohort Study. Fortaleza (CE), Brazil, 2021–2022.

Characteristics	No violence	Interrupted violence	Started violence	Persistent violence	p-value
	n (%)	n (%)	n (%)	n (%)	
Domestic violence	219 (68,0)	25 (7.8)	43 (13.4)	35 (10.9)	
Maternal age (years)					
18–29	106 (63,5)	12 (7.2)	27 (16.2)	22 (13.2)	0.320
30–39	94 (71,6)	10 (7,6)	15 (11.5)	12 (9,2)	
40–49	19 (79,2)	3 (7,8)	1 (4,2)	1 (4.2)	
Marital status					
Single	81 (62.8)	11 (8.5)	21 (16.3)	16 (12.4)	0,144
Stable union	46 (63.9)	3 (4.2)	15 (20.8)	8 (11.1)	
Married	80 (76.2)	9 (8.6)	7 (6.7)	9 (8.6)	
Living with partner					
Yes	166 (69.8)	17 (7.1)	29 (12.2)	26 (10.9)	0.615
No	53 (63.1)	8 (9.5)	14 (16.7)	9 (10.7)	
Maternal school level					
Uncomplete elementary	13 (40.6)	3 (9.4)	6 (18.8)	10 (31.3)	0,050
Complete elementary	21 (56.8)	4 (10.8)	6 (16.2)	6 (16.2)	
High school	134 (70.5)	15 (7.9)	25 (13.2)	16 (8.4)	
University degree	51 (81.0)	3 (4,8)	6 (9.5)	3 (4.8)	
Working mother					
Yes	113 (68.1)	17 (10,2)	17 (10.2)	19 (11.5)	0,149
No	106 (68.0)	8 (5.1)	26 (16.7)	16 (10.3)	
Maternal commom mental disorder					
Yes	34 (51.5)	4 (6.1)	10 (15.2)	18 (27.3)	<0.001
No	185 (72.3)	21 (8.2)	33 (12.9)	17 (6.6)	
Food insecurity (FI)					
No FI	103 (74.6)	12 (8.7)	13 (9.4)	10 (7,3)	0.004
Mild FI	95 (66.9)	12 (8.5)	19 (13.4)	16 (11.3)	
Moderate/severe FI	21 (50,0)	1 (2.4)	11 (26.2)	9 (21.4)	
Head of family					
Mother	40 (49.4)	13 (16.1)	14 (17.3)	14 (17.3)	0.009
Partner	153 (75.4)	8 (3.9)	24 (11.8)	18 (8.9)	
Grandparent	20 (64.5)	4 (12.9)	5 (16,1)	2 (6.5)	
Head of family’s schooling					
Uncomplete elementary	39 (52.7)	6 (8.1)	11 (14.9)	18 (24.3)	0.006
Complete elementary	43 (69.4)	6 (9.7)	10 (16.1)	3 (4.8)	
High school	115 (72.8)	13 (8.2)	20 (12.7)	10 (6.3)	
University degree	22 (84.6)	0 (0.0)	2 (7.7)	4 (15.4)	
Cash transfer					
Yes	130 (63,1)	19 (9.2)	29 (14.1)	28 (13.6)	0.052
No	89 (76.7)	6 (5.2)	14 (12.1)	7 (6.0)	
Monthly family income (minimum wage)					
<1	33 (53.2)	6 (9.7)	13 (21.0)	10 (16.1)	0,245
1–2	125 (68.7)	14 (7.7)	22 (12.1)	21 (11.5)	
3–4	46 (80.7)	4 (7.0)	5 (8.8)	2 (3.5)	
5 or +	14 (73.7)	1 (5.3)	2 (10.5)	2 (10.5)	

The adjusted logistic regression multinomial analysis estimated the odds ratio of mothers who stopped, started, and persistently reported DV, using mothers who did not report DV as the reference category, identifying their associated risk factors (Table 5). Maternal CMD was strongly associated with persistent DV, as mothers with this condition had a 4-fold higher risk of reporting DV as compared to mothers with no report of DV. Severe to moderate food insecurity remained associated only with starting DV; the mother being the head of the family was strongly associated with all three patterns of DV, suggesting a possible gender issue be explored; head of family’s poor schooling presented an almost three times higher risk of continuous DV as compared to families with no report of VD. Partner living at home was identified as a protective factor, but was not statistically significant at the 5% level.

**Table 5:** Multinomial logistic regression model of factors associated with patterns of domestic violence. Iracema-COVID Cohort Study. Fortaleza (CE), Brazil, 2021–2022.

Characteristics*	Stop DV		Start DV		Persistent DV	
	aOR^†^ 95%CI	p-value	aOR^†^ 95%CI	p-value	aOR^†^ 95%CI	p-value
Maternal CMD	0.97 0.30–3.14	0.965	1.28 0.55–2.95	0.567	4.71 2.11–10.48	<0.001
Moderate or severe food insecurity	0.33 0.04–2.68	0.298	2.77 1.16–6.61	0.022	1.86 0.68–5.08	0.223
Mother as head of the household	8.10 2.85–23.00	<0.001	2.47 1.01–6.02	0.047	5.29 1.94–14.39	0.001
Head of household with elementary schooling	2.46 1.00–6.05	0.050	1.54 0.75–3.13	0.237	2.89 1.26–6.63	0.012
Biological father living at home	0.49 0.17–1.47	0.209	0.89 0.38–2.13	0.809	0.40 0.14–1.16	0.093

*No DV was the reference category;

^†^Adjusted odds ratio (aOR). The model also included family monthly income, cash transfer, marital status, maternal work, schooling, and age.

## DISCUSSION

This study follows a cohort of mothers who gave birth in public health facilities during the first wave of the COVID-19 pandemic, from July to August 2020. Domestic violence, irrespective of type or severity, was reported by 19 and 24% of mothers at 18 and 24 months after birth, respectively, with a recall period of six months. This prevalence rates are quite higher than the 3.3% observed by 12–18 months postpartum in Sweden^8^, and the 1% of domestic physical abuse among mothers two years post childbirth in the USA^13^, but considerably lower than rates of postpartum DV found in low-income countries, such as 34–35% in Bangladesh^9,14^ and 59–65% in Ethiopia^5,6^.

In our study, the most frequent types of DV reported by mothers in the postpartum period were: verbal aggression (20%), drunkenness and/or illicit drug abuse (5%), threats of expelling from the home (3%), and physical aggression (1.6%). The proportion of mothers who reported two or more of these types of harassment increased from 2% by 18 months to 12% by 24 months postpartum. Several studies on DV have also identified this kind of physical and psychological abuse, but some have specified other gruesome forms such as keeping women at a distance, not purchasing home’s fundamental needs, and being physically forced to engage in a sexual act^5,15^.

Our data suggest that DV increased progressively during the postpartum period. While by 18 months 2% of the mothers reported two or more types of DV, by 24 months such proportion rose to 12%, showing a substantial increase in the period. We did not find studies that measured DV in various moments within the postpartum period. Most of them measured DV during pregnancy and postpartum, showing that in high-income countries, such as Sweden, DV rose from 2.5% in pregnancy to 3.3% in the postpartum period^8^ and Quebec, Canada, where qualitative data reveal escalating violence from before conception, during pregnancy and two years after birth^16^. In São Paulo, Southeastern Brazil, 2.3 and 5.3% of women disclosed physical violence during pregnancy and by 12 months postpartum, respectively^17^. Data from low-income countries, however, have shown a reverse trend, with extremely high rates of DV during pregnancy, ranging from 35% in Nepal to 59% in Ethiopia, with some progressive rate reduction during the postpartum period^6,18^.

From a longitudinal perspective, DV was persistently present in 11% of homes during the second year period postpartum, while 68% were free of any form of DV, according to the mother’s report. Among mothers who experienced changes in the pattern of DV, 8% reported cessation of DV at home, while 13% were exposed to a DV ramping up. Among these households that experienced changes in the pattern of DV, there was a 4.98% increase (p=0.048) in the direction of starting DV, considering the mothers’ reports from the 18 months to the 24 months survey round. We did not find other studies with such data to compare.

Maternal and family factors identified as associated with reported DV in the adjusted analysis were: maternal CMD, moderate to severe FI, family headed by the mother and head of family’s poor schooling. Biological father living at home was identified as a protective factor, but was only near statistical significance. Mothers with CMD presented almost five-times higher probability of persistent DV as compared to mothers with no report of DV in the period. Similarly, data from a meta-analysis study found 2.8 and 4.1 greater odds of DV among women with depressive and anxiety disorders, respectively, compared to women without mental disorders^19^. Data from another meta-analysis of longitudinal data on the specific association between DV and perinatal mental disorders estimates a 3-fold increase in the odds of severe depressive symptoms in the postnatal period, after exposure to partner violence during pregnancy^20^. Several other studies pointed out DV and other forms of violence against women as associated with increased risk of mental disorder^8,15,21,22^. Some studies have shown, however, that the cause of violence is just the woman’s poor mental health condition^23^. Hence, it is sometimes difficult to establish which is cause and effect, but probably there is feedback.

Families with severe to moderate FI were more prone to come from families of starting a DV pattern, as compared to those families experiencing mild FI or food security, suggesting that the onset of a hunger situation may be a trigger to DV. Thus, food insecurity might serve as a warning for the investigation/suspicion of DV. Other studies in Brazil have identified FI as a major risk factor for DV, suggesting that the psychosocial aspect of the families should be considered when implementing interventions to reduce household FI^24^. Worldwide, a study in Nepal identified FI associated with increased odds of emotional and physical, but not sexual violence^25^. Also, in California, USA, in African-American households exposed to severe FI, women were more likely to report serious partner violence^26^.

In our study all three family patterns of DV: interrupted, started, and persistent, were more common in families headed by the mother than in households headed by the partner or grandparents. Data from low and middle-income countries (LMIC) show that DV was strongly associated with women’s decision-making power^6,7,9^, with behavioral change communication interventions being suggested to enhance mother’s decision-making acceptance^9^. Thus, policies of women’s empowerment advocacy, without a concomitant movement to promote behavioral changes in society, such as reducing *machismo* or gender issues, should be seen with caution, as they may instigate instead of mitigate DV. The United Nations’ Sustainable Development Goal 5 aims to “achieve gender equality and empower all women and girls, which is critical to reducing violence against women”^27^.

Households whose head had poor schooling were significantly associated with persistent DV, with one out of four families being headed by the mothers. Low education status of husbands, partners, and also mothers has been found as a recurrent determinant factor of DV in several LMIC^5,7,18^.

The main strength of this paper is its longitudinal design. Women were asked about the occurrence of DV on two occasions, with a recall period of six months on each, while in most studies the occurrence of DV is measured at least once in a lifetime or the previous year. While studies that use DV cases registered in health facilities or police stations work with more severe cases, showing greater specificity, our cases are more comprehensive in terms of severity, showing greater sensitivity. Among the limitations of the study is the fact that we did not use a validated scale, a common fact among DV studies, which usually employ a wide variety of instruments, which makes it difficult to compare the results. Also, because the interviews were conducted face-to-face at home, this may have inhibited the interviewee from positively answering certain questions, which may render our estimates conservative.

In conclusion, policies for prevention and control of DV have little chance of effective success if they do not rely on interventions in the three following areas identified in this study:

a) Health — improving women’s mental health care, identifying women with CMD in the primary health care level, and considering them as suspected cases of DV;
b) Nutrition — assisting families in a condition of food insecurity, especially in the moderate and severe forms, implementing means of screening in the community;
c) Education — fostering the educational level of young people of both sexes, investing on the effort to make them reach at least high school education.
